# Differential Effects of Pathological Beta Burst Dynamics Between Parkinson’s Disease Phenotypes Across Different Movements

**DOI:** 10.3389/fnins.2021.733203

**Published:** 2021-11-11

**Authors:** Raumin S. Neuville, Matthew N. Petrucci, Kevin B. Wilkins, Ross W. Anderson, Shannon L. Hoffman, Jordan E. Parker, Anca Velisar, Helen M. Bronte-Stewart

**Affiliations:** ^1^Department of Neurology and Neurological Sciences, Stanford University School of Medicine, Stanford, CA, United States; ^2^School of Medicine, University of California, Irvine, Irvine, CA, United States; ^3^Department of Psychology, University of California, Los Angeles, Los Angeles, CA, United States; ^4^Smith-Kettlewell Eye Research Institute, San Francisco, CA, United States; ^5^Department of Neurosurgery, Stanford University School of Medicine, Stanford, CA, United States

**Keywords:** beta oscillations, Parkinson’s disease (PD), local field potentials (LFP), subthalamic nucleus (STN), deep brain stimulation (DBS), beta bursts, akinetic rigid, tremor dominant

## Abstract

**Background:** Resting state beta band (13–30 Hz) oscillations represent pathological neural activity in Parkinson’s disease (PD). It is unknown how the peak frequency or dynamics of beta oscillations may change among fine, limb, and axial movements and different disease phenotypes. This will be critical for the development of personalized closed loop deep brain stimulation (DBS) algorithms during different activity states.

**Methods:** Subthalamic (STN) and local field potentials (LFPs) were recorded from a sensing neurostimulator (Activa^®^ PC + S, Medtronic PLC.) in fourteen PD participants (six tremor-dominant and eight akinetic-rigid) off medication/off STN DBS during 30 s of repetitive alternating finger tapping, wrist-flexion extension, stepping in place, and free walking. Beta power peaks and beta burst dynamics were identified by custom algorithms and were compared among movement tasks and between tremor-dominant and akinetic-rigid groups.

**Results:** Beta power peaks were evident during fine, limb, and axial movements in 98% of movement trials; the peak frequencies were similar during each type of movement. Burst power and duration were significantly larger in the high beta band, but not in the low beta band, in the akinetic-rigid group compared to the tremor-dominant group.

**Conclusion:** The conservation of beta peak frequency during different activity states supports the feasibility of patient-specific closed loop DBS algorithms driven by the dynamics of the same beta band during different activities. Akinetic-rigid participants had greater power and longer burst durations in the high beta band than tremor-dominant participants during movement, which may relate to the difference in underlying pathophysiology between phenotypes.

## Introduction

Exaggerated resting state beta band (13–30 Hz) oscillations and synchrony are pathophysiological markers of hypokinetic aspects of Parkinson’s disease (PD). When averaged over time, these oscillations appear as elevated portions of the local field potential (LFP) power spectral density (PSD) above the broadband 1/f spectrum (He, 2014; Shreve et al., 2017). Beta band power is attenuated on dopaminergic medication and during subthalamic (STN) deep brain stimulation (DBS); the degree of attenuation has been correlated to the degree of improvement in bradykinesia and rigidity, whereas averaged resting state beta band power is less robustly correlated with PD motor signs (Brown et al., 2001; Cassidy et al., 2002; Levy et al., 2002; Williams et al., 2002; Priori et al., 2004; Kühn et al., 2006, 2008, 2009; Weinberger et al., 2006; Ray et al., 2008; Bronte-Stewart et al., 2009; Eusebio et al., 2011; Whitmer et al., 2012; Quinn et al., 2015; Kehnemouyi et al., 2021).

Recently, it has been shown that physiological resting state beta oscillations are represented by short duration fluctuations in power (beta bursts) in the striatum and cortex of healthy non-human primates (Feingold et al., 2015). These authors suggested that the precise temporal dynamics of beta bursts may be more reliable markers of PD than averaging beta activity over periods of time. Burst dynamics in PD have been studied during rest (Tinkhauser et al., 2017; Anderson et al., 2020), but less is known about real time beta burst dynamics during movement and whether beta burst dynamics differ during fine motor or limb movements and/or during gait and freezing of gait (FOG) (Anidi et al., 2018; Lofredi et al., 2019; Kehnemouyi et al., 2021). The duration of beta bursts is a relevant neural control variable for closed loop DBS systems, which can precisely target (shorten) the duration of beta bursts, but it is not known how this variable may change among movements which may necessitate a different response from a closed-loop algorithm (Petrucci et al., 2020a).

In addition to differences among tasks, it is unclear how beta burst dynamics may differ between sub bands of beta or between Parkinson’s disease phenotypes. Previous studies that have evaluated phenotype differences primarily focused on high (20–35 Hz) and low (10–20 Hz) beta band power in the operating room or perioperative state (i.e., the week after implantation). Differences in high beta band power were demonstrated between tremor-dominant (TD) and akinetic-rigid (AR) phenotypes at rest, but not during movement in an elbow-flexion task in the operating room (Godinho et al., 2021). Furthermore, within band differences between rest and movement were observed for each phenotype (low beta for tremor-dominant and high beta for akinetic-rigid). Differences in resting state high beta power have also been reported in the immediate post-operative period between people with and without FOG, as assessed off medication in the pre-operative period (Toledo et al., 2014). To date, no study has compared burst durations within sub bands of beta, between disease phenotypes, and during different movements using a chronically implanted device. In this study, we investigated whether beta band peak frequencies were conserved or were different during fine, limb, and/or axial movements in people with PD, and whether there were differences in beta band and sub band power and burst dynamics between the akinetic-rigid and tremor-dominant phenotypes.

## Materials and Methods

### Human Participants

**FIGURE 1:**
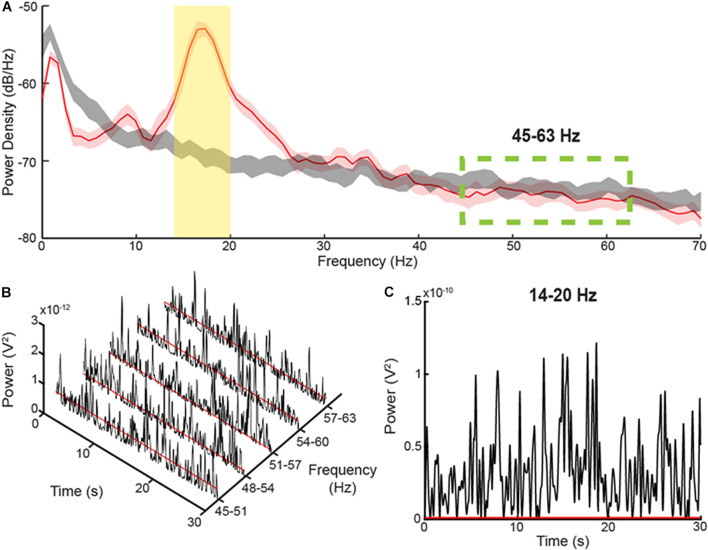
Method for determining burst durations **(A)** PSD diagrams of 30 s during a Parkinsonian resting state (red) versus pink noise (gray), which can be considered simulated 1/f baseline activity in the brain. The yellow, shaded area represents the 6 Hz band centered on the peak of the PSD. The green-dashed lines display the area where there is no elevation of the resting state PSD above the pink noise simulated 1/f activity. **(B)** Consecutive, 6 Hz envelopes of the filtered and squared resting state LFP during the resting state within the non-pathological, high frequency range. The red lines signify the median power of the troughs from each envelope. **(C)** The envelope of a 6 Hz band within the elevated area of the PSD diagram. The threshold for determining burst durations, represented by the red line, was calculated by multiplying the average median trough powers within the high frequency range by a factor of four.

Fourteen participants (10 male) with clinically established PD underwent bilateral implantation of DBS leads (model 3389, Medtronic PLC., Minneapolis, MN, United States) in the sensorimotor region of the subthalamic nucleus (STN) using a standard functional frameless stereotactic technique and microelectrode recording (MER) (Brontë-Stewart et al., 2010; Quinn et al., 2015). Long-acting dopaminergic medication was withdrawn over 24 h (72 h for extended-release dopamine agonists) and short-acting medication was withdrawn for over 12 h before surgery and before each study visit. One participant took an extra short-acting carbidopa/levodopa tablet 5 h before the experiments and was included as their resting state LFP spectra were similar 6.25 and 8.5 h later, suggesting resolution of an attenuating effect of medication on beta power (Trager et al., 2016). The preoperative selection criteria and assessment of participants have been previously described (Taylor Tavares et al., 2005; Bronte-Stewart et al., 2009; de Solages et al., 2010). The dorsal and ventral borders of each STN were determined using MER, and the base of electrode 0 of the Medtronic 3389 lead was placed at the MER defined ventral border of the STN (Marceglia et al., 2006; de Solages et al., 2010, 2011). The DBS leads were located in the STN (Figure 1A). All participants signed a written consent and the study was approved by the Food and Drug Administration, Investigational Device Exemption and the Stanford School of Medicine Institutional Review Board. Each participant was classified as TD or AR phenotype based on previously described criteria (Quinn et al., 2015; Trager et al., 2016; Shreve et al., 2017) and the more and less affected sides were determined by unilateral Unified Parkinson’s Disease Rating Scale (UPDRS) part III sub-scores.

### Experimental Protocol

**FIGURE 2:**
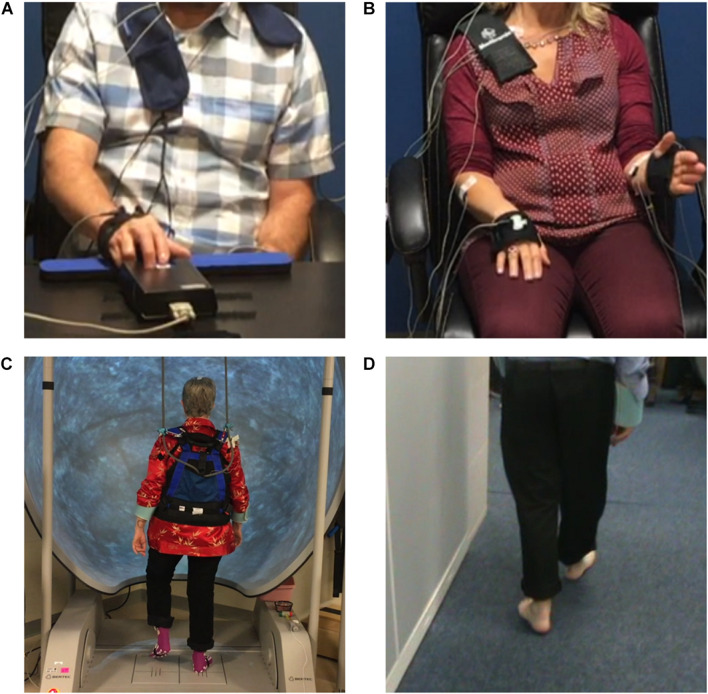
The **(A)** quantitative digitography (QDG), **(B)** instrumented repetitive wrist-flexion extension (WFE), **(C)** stepping in place (SIP), and **(D)** forward walking (FW) tasks.

All experiments were performed within 2 months after DBS lead placement in the off medication/off DBS state. Recordings were collected in the Stanford Human Motor Control and Neuromodulation Laboratory. Experiments started with a resting state recording, during which each participant sat still for 30 s. Participants completed four different movement tasks (Figure 2): (1) quantitative digitography (QDG) on an engineered keyboard (Bronte-Stewart et al., 2000; Taylor Tavares et al., 2005; Trager et al., 2015) (2) instrumented repetitive wrist-flexion extension (WFE) (Koop et al., 2006, 2008; Louie et al., 2009), (3) stepping in place (SIP) on dual force plates (Nantel et al., 2011), and (4) free walking (FW). During the QDG task, participants were seated with their elbow flexed at approximately 90° and the wrist was supported by a pad alongside a customized engineered keyboard. Visual and auditory feedback was minimized, as the participants had their eyes closed and wore headphones that played white noise to limit auditory feedback from the key tapping. With the index and middle fingers placed on individual keys, participants were instructed to tap each key in an alternating pattern as fast and regularly as possible for 30 s. For the instrumented rWFE task, participants were seated with their elbow flexed at approximately 90° and the hand in the mid-pronated-supinated position before they were asked to flex and extend their wrists as fast as possible for 30 s. During the SIP task, participants were instructed to perform alternating stepping on dual force plates for 100 s. For the FW task, all participants walked for approximately 1 min within a lab space that consisted of circular and straight paths (two participants only walked within the straight path) with interspersed 90–180° turns. All movements were self-paced.

### Data Acquisition and Analysis

Local field potentials (LFPs) from the STN were recorded from the electrode pair of the DBS lead that had the greatest resting state beta band peak power and the least artifact (electrode pairs 0–2 or 1–3 of the Medtronic 3389 lead; Supplementary Table 1). The pre-amplified LFP was high-pass filtered at 2.5 Hz and low-pass filtered at 100 Hz. LFP data was sampled at a rate of 422 Hz (10-bit resolution). The gains used for the experiments were set at 2,000, and since these experiments were off stimulation, we set the center frequency of the Activa^®^ PC + S neurostimulator to the lowest frequency setting of 2.5 Hz (Blumenfeld et al., 2017). The uncompressed LFP data were extracted via telemetry using the Activa^®^ PC + S tablet programmer and then transferred to a computer for offline analysis in MATLAB (version 9.5, The MathWorks Inc., Natick, MA, United States). LFP data used for analysis was from the first 30 s of movement or from the maximum length of continuous movement without cueing. The power spectra were estimated using Welch’s method, which used a 1-s Hanning window with 50% overlap (Welch, 1967). The peak frequency in the beta band was detected using a peak detection algorithm (de Solages et al., 2010). The peak detection algorithm runs through each point in the PSD under 40 Hz and labels a peak when both the central and lower frequency, adjacent bin are greater than the mean of the 3rd to 5th bins in the direction of lower frequency and the central and higher frequency, adjacent bin are greater than the mean of the 3rd to 6th bins in the direction of higher frequency. The chosen peak was then verified by visual inspection. If more than one peak was detected, the peak with the greatest power was chosen. In two movement episodes, the algorithm failed to detect a peak, which was evident on visual inspection.

### Local Field Potential Burst Dynamics Determination

The method for determining the burst dynamics was adopted from Anderson et al.(2020; Figure 1), which uses a baseline threshold calculated from a portion of the PD LFP spectrum that corresponds to the power and burst dynamics of a simulated, physiological 1/f spectrum. The baseline method captures a broader range of beta burst durations than high power burst detection methods. The LFP within the band of interest was first filtered using a 6-Hz bandwidth, zero-phase 8th order Butterworth filter, and then squared. An envelope was formed by interpolating between the consecutive peaks of the filtered, squared signal, Figure 1C. The threshold for characterizing individual bursts is calculated by averaging the median trough amplitudes from 5 consecutive overlapping 6 Hz bands in the 45–63 Hz PD gamma spectrum and multiplying the median trough power by a factor of four, Figures 1B,C. The 5 overlapping bands used to define the threshold were set to the following frequencies: 45–51 Hz, 48–54 Hz, 51–57 Hz, 54–60 Hz, and 57–63 Hz. In contrast to the elevated, beta frequency band of the PD spectrum, the higher frequency band (45–63 Hz) is not elevated above the physiological LFP activity or 1/f signal, and contains burst dynamics resembled that of physiological neural activity (He, 2014; Anderson et al., 2020). Burst duration was calculated as the time between consecutive crossings of the envelope across the baseline threshold. The average power of each burst was also calculated (mean burst power) by averaging the power envelope between consecutive crossings across the baseline threshold.

### Statistics

The primary outcome variables were peak frequency during movement, power, mean burst power, and mean burst duration. Power, mean burst power, and mean burst duration were calculated separately for low beta (14–20 Hz) and high beta (22–28 Hz) frequency bands. We used 6 Hz bands to allow for equal comparison of burst durations between bands (Anderson et al., 2020). Normalization of all power values was completed through division by the average power of the squared signal in the 45–63 Hz frequency band during the resting state; this high frequency band overlaps with the physiological 1/f curve and is clear of elevated, Parkinsonian beta activity (Anidi et al., 2018). Independent *t*-tests were used to compare age, disease duration, and pre-operative UPDRS scores between the TD and AR phenotypes. One-way repeated measures ANOVAs compared peak frequencies in the PSDs and variation in power and burst metrics among the different movement tasks in high and low beta with each STN treated individually and PD phenotype as a between-subjects factor. Analyses were corrected for multiple comparisons using Bonferroni correction. In the presence of a violation of Mauchly’s test of sphericity, the Greenhouse–Geisser correction was applied. There was one trial per movement task for each participant.

## Results

**FIGURE 3:**
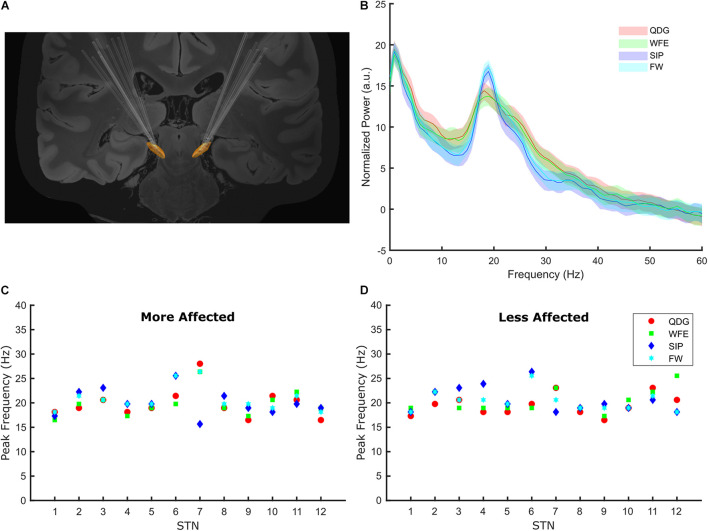
**(A)** The lead placements for all participants for the left and right STNs. **(B)** The normalized grand average power spectral density plots for the four tasks. The power spectra were normalized to the average power in 45–63 Hz. The grand average PSD for each task was generated using the averaged data across all STNs during 30 s of movement. **(C,D)** The beta frequency peaks for each task by participant in the more and less affected STNs.

Of the 14 participants, six were classified as TD and eight were classified as AR. The age of the group (mean ± SD) was 57.0 ± 10.2 years (TD 60.4 ± 10.8 years; AR 54.4 ± 9.5 years), and the disease duration from symptom onset was 7.7 ± 3.7 years (TD 8.7 ± 4.3 years; AR 7.0 ± 3.2 years). UPDRS III scores (mean ± SD) in the pre-operative off- and on-medication state were 39.2 ± 14.8 (TD 44.7 ± 12.3; AR 37.6 ± 16.1) and 23.2 ± 14.1 (TD 19.2 ± 7.7; AR 25.4 ± 16.5), respectively. There were no significant differences in age, disease duration, or pre-operative UPDRS scores between participants classified as TD and AR (*p* > 0.05). The DBS leads were well placed within the STN, Figure 3A.

### Peak Frequency Was Conserved Across Different Movements

Among the cohort of 24 STNs (8 TD and 16 AR) for whom peaks could be detected, peaks of elevated beta power were detected in 98% (94/96) of all movement episodes during the different tasks across all STNs, demonstrating that exaggerated beta band oscillations and synchrony were present during fine motor, limb and axial movements. In two TD participants, no peak was detected in either hemisphere, so they were excluded from this analysis. Beta peaks across the four movement tasks is depicted in the grand average PSDs in Figure 3B. The peak frequency did not differ across the movement tasks [*F*(1.62,35.53) = 0.58, *p* = 0.53, partial η^2^ = 0.026) or between phenotypes [*F*(1,22) = 0.39, *p* = 0.54, partial η^2^ = 0.017), and there was no interaction between task and phenotype [*F*(1.62,35.53) = 2.93, *p* = 0.076, partial η^2^ = 0.12) on peak frequency. Peak frequency across movements was similar in the more (Figure 3C) and less affected (Figure 3D) STNs.

### Differences in Power Between Tremor-Dominant and Akinetic-Rigid Groups in the High Beta Band

**FIGURE 4:**
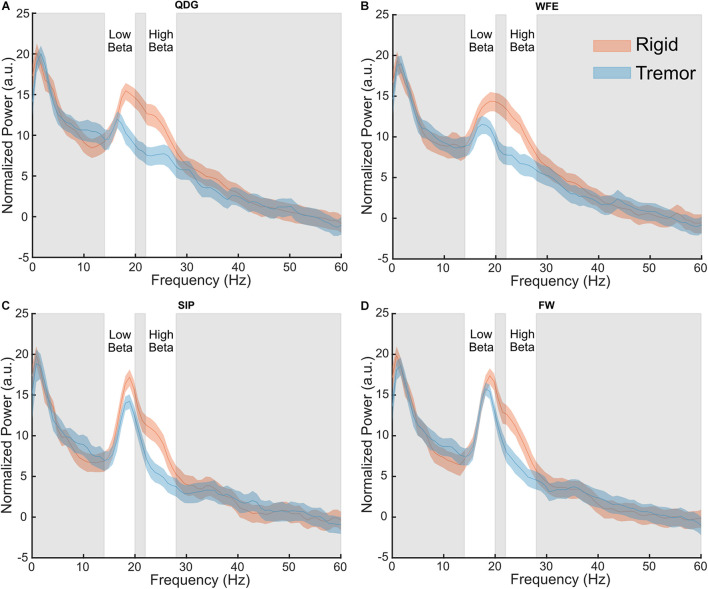
Grand average normalized power spectral density plots for each phenotype in the **(A)** QDG, **(B)** WFE, **(C)** SIP, and **(D)** FW tasks. There were significant differences (*p* < 0.05) between phenotypes in the high beta band.

**FIGURE 5:**
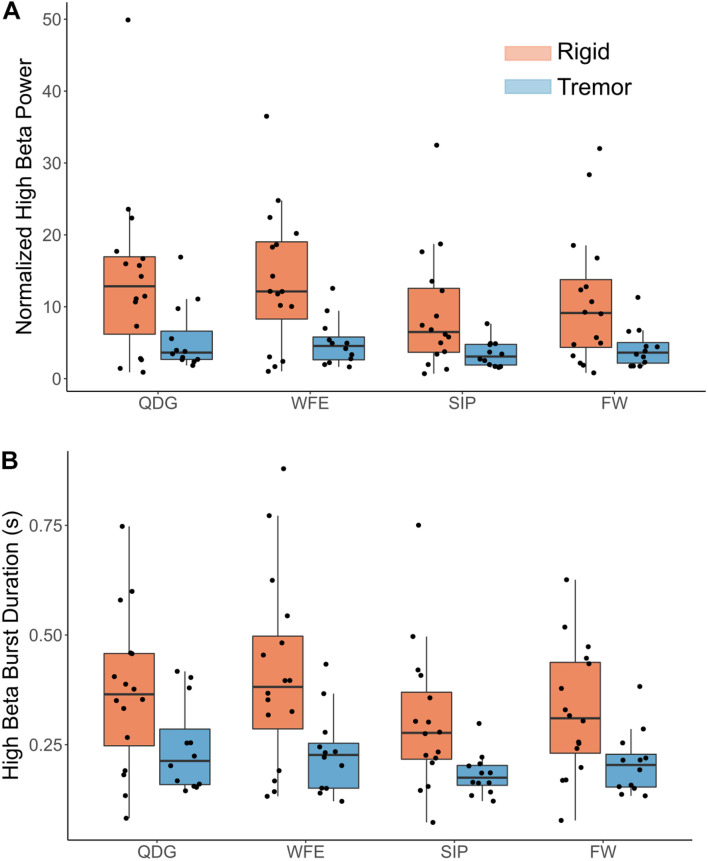
**(A)** Normalized high beta peak power and **(B)** high beta burst durations by task and phenotype. Note, a significant main effect was observed between groups (*p* < 0.05) but not task, and there was no significant interaction effect.

Normalized power was analyzed across movement tasks and between the TD and AR groups in high and low beta for the full cohort of 28 STNs (Figure 4). In high beta, there was a significant effect of phenotype [*F*(1,26) = 8.84, *p* = 0.006, partial η^2^ = 0.25], but not of task [*F*(1.33,34.62) = 2.93, *p* = 0.085, partial η^2^ = 0.10] (Figure 4). Normalized high beta power was greater for the AR group compared to the TD group across all movements (Figure 5A). In low beta, there were no differences in normalized power between phenotypes [*F*(1,26) = 1.27, *p* = 0.270, partial η^2^ = 0.047] or across tasks [*F*(1.39,36.18) = 0.88, *p* = 0.39, partial η^2^ = 0.033]. There was also no interaction between task and PD phenotype for normalized power in either low beta [*F*(1.39,36.18) = 0.088, *p* = 0.85, partial η^2^ = 0.003] or high beta [*F*(1.33,34.62) = 0.59, *p* = 0.49, partial η^2^ = 0.022].

### Differences Between the Akinetic-Rigid and Tremor-Dominant Groups and Across Tasks in the High Beta Band

Mean burst duration was analyzed across movement tasks and between the TD and AR groups in high and low beta for 27 STNs (11 TD and 16 AR) (Figure 5B). Burst data for one STN of a TD patient was excluded because burst duration in low beta during the FW task was identified as a statistical outlier (greater than 3 SD from the mean). In high beta, there was both a significant effect of phenotype [*F*(1,25) = 8.92, *p* = 0.006, partial η^2^ = 0.26] and of task [*F*(1.75,43.67) = 4.48, *p* = 0.021, partial η^2^ = 0.15]. High beta mean burst duration was greater for the AR phenotype compared to the TD phenotype across all movements (Figure 5B). Pairwise comparisons between movement tasks did not reveal significant differences between specific tasks with the Bonferroni correction (*p* > 0.05). In low beta, there were no differences in mean burst duration between phenotypes [*F*(1,25) = 2.34, *p* = 0.14), partial η^2^ = 0.085] or across tasks [*F*(1.36,33.87) = 0.18, *p* = 0.75, partial η^2^ = 0.007]. There was no interaction between task and PD phenotype for mean burst duration for either low beta [*F*(1.36,33.87) = 0.44, *p* = 0.57, partial η^2^ = 0.017] or high beta [*F*(1.75,43.67) = 0.43, *p* = 0.63, partial η^2^ = 0.017].

### Differences in Mean Burst Power Between Parkinson’s Disease Phenotypes, but Not Across Tasks, in High Beta

Mean burst power was analyzed across movement tasks and between PD phenotypes in high and low beta (Figure 5A). In high beta, there was a significant effect of phenotype [*F*(1,25) = 9.06, *p* = 0.006, partial η^2^ = 0.266], but no effect of task [*F*(1.34,33.50) = 0.41, *p* = 0.59, partial η^2^ = 0.016]. High beta mean burst power was greater for the AR phenotype compared to the TD phenotype across all movements. In low beta, there were no differences in mean burst power between phenotypes [*F*(1,25) = 3.12, *p* = 0.090, partial η^2^ = 0.11] or across tasks [*F*(1.30,32.52) = 0.24, *p* = 0.69, partial η^2^ = 0.010]. There was no interaction between task and PD phenotype for mean burst power in both low [*F*(1.30,32.52) = 0.084, *p* = 0.84, partial η^2^ = 0.003] and high [*F*(1.34,33.50) = 0.040, *p* = 0.90, partial η^2^ = 0.002] beta.

## Discussion

The results of this study demonstrate that pathological beta oscillations and synchrony are present during ongoing movement and that the frequencies of the beta band peak were similar among fine, limb and axial movements. However, people with PD classified as akinetic-rigid showed greater high beta power and high beta burst duration and burst power across all tasks compared to those classified as tremor-dominant. This difference may point to an important difference in pathophysiology between phenotypes.

### The Clinical Significance of the Conservation of Beta Band Peak Frequency Across Movements

Several studies have demonstrated that beta power decreased before, at the onset of, and during movement in human participants with PD and in non-human primates (Kühn et al., 2004; Litvak et al., 2011; Joundi et al., 2013; Johnson et al., 2016; Blumenfeld et al., 2017; Syrkin-Nikolau et al., 2017; Anidi et al., 2018; Fischer et al., 2018; Hell et al., 2018; Lofredi et al., 2019). This has led to a frequent generalization in the literature that beta power “goes away” during movement. The results of this study demonstrate that beta peaks were still evident during movement, and that the peak frequencies were conserved among fine motor and limb movements and during gait. This may alleviate concerns regarding the implementation of closed loop DBS in freely moving people. Up to now, closed loop DBS classifier algorithms have used estimates of resting state beta band power as the control variable (Little et al., 2013, 2016a,b; Rosa et al., 2015, 2017; Piña-Fuentes et al., 2017, 2019; Afzal et al., 2019; Velisar et al., 2019; Petrucci et al., 2020b). Such algorithms require knowledge of the peak frequency of the band of interest and until now it was not known whether the same beta band could be used to drive closed loop DBS when the person is working at their computer, eating, dressing, or when walking. Although others have seen that there was a slight shift in peak frequency between different motor states (Canessa et al., 2020), we observed no significant difference across four different tasks. The conservation of the choice of the band of interest (determined by the peak frequency) among fine, limb, and axial movements suggests that the same classifier algorithms will be appropriate across movement states. Additionally, even if small differences are observed in peak frequencies, most current methods for tracking beta band look across a bandwidth of 6 ± Hz and therefore are robust against shifts in peak frequencies that still fall within these bandwidths (Afzal et al., 2019; Velisar et al., 2019; Petrucci et al., 2020a).

### Differences in Pathophysiology Between Motor Phenotypes

Our results demonstrate that the AR group shows greater high beta power and burst metrics across tasks compared to the TD group. This is the first study to show neural oscillatory differences in the STN between PD phenotypes across different movement states. High beta oscillations in the STN have been posited to relate to STN-cortical connections in PD, whereas low beta oscillations relate to intrinsic pathophysiology within the basal ganglia (Oswal et al., 2020). Specifically, coupling in high beta between the STN and supplementary motor area (SMA) correlates with fiber density between those two regions. Furthermore, improvement in rigidity with DBS has been shown to be related to connectivity to the SMA (Akram et al., 2017). The differences observed in our study between AR and TD may reflect differences in these STN-cortical interactions. This is further supported by the previous work demonstrating greater high beta power in freezers compared to non-freezers (Toledo et al., 2014) and tremor-dominant vs. akinetic-rigid (Godinho et al., 2021) at rest. Together, these results point to a pathological low beta oscillation that is consistent across phenotypes and then a potentially separate high beta oscillation that may be more specific to akinetic-rigid symptoms regardless of task. These differences could be utilized to improve patient-specific closed-loop loop algorithms due to recent advances in technology (Summit^TM^ RC + S, Medtronic PLC.) that can track multiple bands simultaneously.

## Limitations

Due to the limited number of investigative devices (Activa^®^ PC + S, Medtronic PLC., Minneapolis, MN, United States) allocated to centers, the sample size was small but comparable to previous studies (Quinn et al., 2015; Blumenfeld et al., 2017; Syrkin-Nikolau et al., 2017; Anidi et al., 2018). Additionally, the tremor-dominant cohort displayed a mix of presence versus absence of tremor across the tasks and therefore it is difficult to say with certainty that the observed differences in high beta are a phenological difference between phenotypes or that the action of the tremor itself is specific to high beta. We did confirm in at least 2 participants that there was not an appreciable difference in high beta during the tremor and non-tremor periods when tremor arose in the middle of the trial (see Supplementary Figures 1–8). A larger cohort of tremor-dominant participants is needed to confirm these findings.

## Conclusion

The results of this study demonstrated that exaggerated beta power was evident during fine motor, limb and axial movements and that the peaks of the frequency band of elevated power were similar during such different movements. Furthermore, there were significant differences in beta power and burst durations between the akinetic-rigid and tremor-dominant phenotypes in the high beta, but not low beta. These findings are critical for future closed loop DBS systems, which will require an input that is both indicative of the disease state as well as robust through the patient’s activities of daily living.

## Data Availability Statement

The raw data supporting the conclusions of this article will be made available by the authors, without undue reservation.

## Ethics Statement

The studies involving human participants were reviewed and approved by the Stanford School of Medicine Institutional Review Board. The patients/participants provided their written informed consent to participate in this study.

## Author Contributions

RN: conceptualization, methodology, software, validation, formal analysis, investigation, data curation, writing – original draft and review and editing, visualization, supervision, and project administration. MP: conceptualization, methodology, validation, formal analysis, writing – review and editing, and supervision. KW: formal analysis, writing – review and editing, and visualization. RA: methodology, software, validation, formal analysis, investigation, writing – original draft and review and editing, visualization, and supervision. SH: formal analysis, writing – review and editing, and visualization. JP: methodology and validation. AV: software and investigation. HB-S: conceptualization, methodology, writing – original draft and review and editing, supervision, and funding acquisition. All authors contributed to the article and approved the submitted version.

## Conflict of Interest

HB-S is a member of the Medtronic PLC., Clinical Advisory Board. The remaining authors declare that the research was conducted in the absence of any commercial or financial relationships that could be construed as a potential conflict of interest.

## Publisher’s Note

All claims expressed in this article are solely those of the authors and do not necessarily represent those of their affiliated organizations, or those of the publisher, the editors and the reviewers. Any product that may be evaluated in this article, or claim that may be made by its manufacturer, is not guaranteed or endorsed by the publisher.
