# Supplementary material for: Differential Effects of Pathological Beta Burst Dynamics Between Parkinson’s Disease Phenotypes Across Different Movements
Source: Front Neurosci. 2021 Nov 11;15:733203. doi: 10.3389/fnins.2021.733203 (PMC8631908; doi:10.3389/fnins.2021.733203)
Supplement: Supplementary file 1 [file Image_1.pdf]

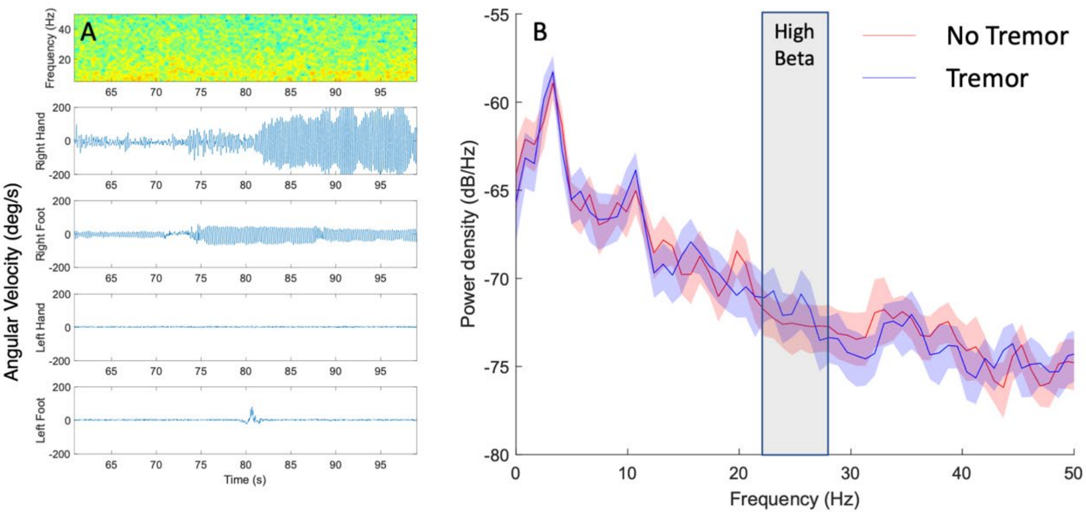

Figure S1. Example figure of Participant 3 (tremor-dominant) during the QDG task (right hand, left STN). **(A)** Synchronized spectrogram of neural data and movement of each limbed measured by a gyroscope. **(B)** Power spectral density plots of periods of no tremor and tremor in the right hand.

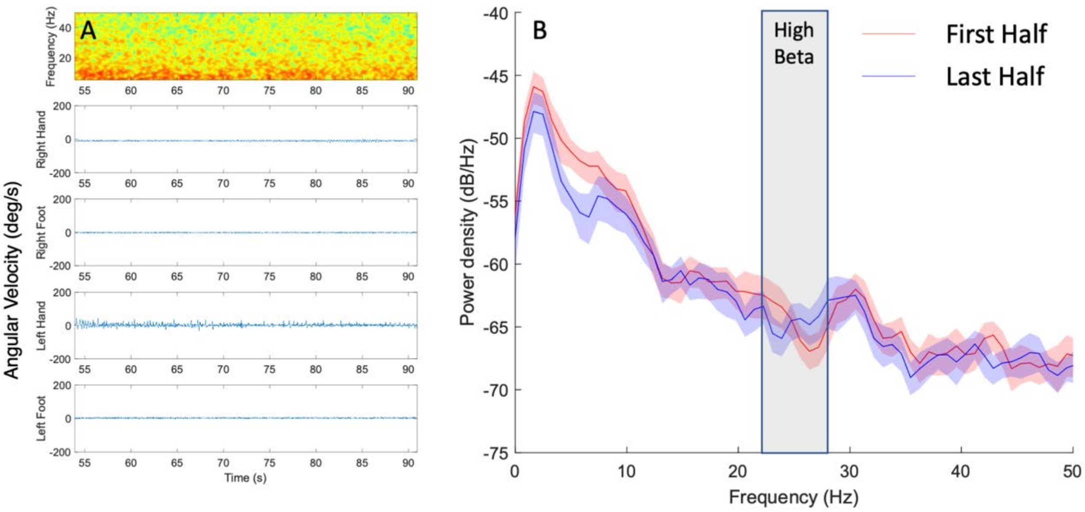

Figure S2. Example figure of Participant 3 (tremor-dominant) during the QDG task (left hand, right STN). **(A)** Synchronized spectrogram of neural data and movement of each limbed measured by a gyroscope. **(B)** Power spectral density plots of the first and last half of trial as there was no onset of tremor in the middle of the trial for the left body.

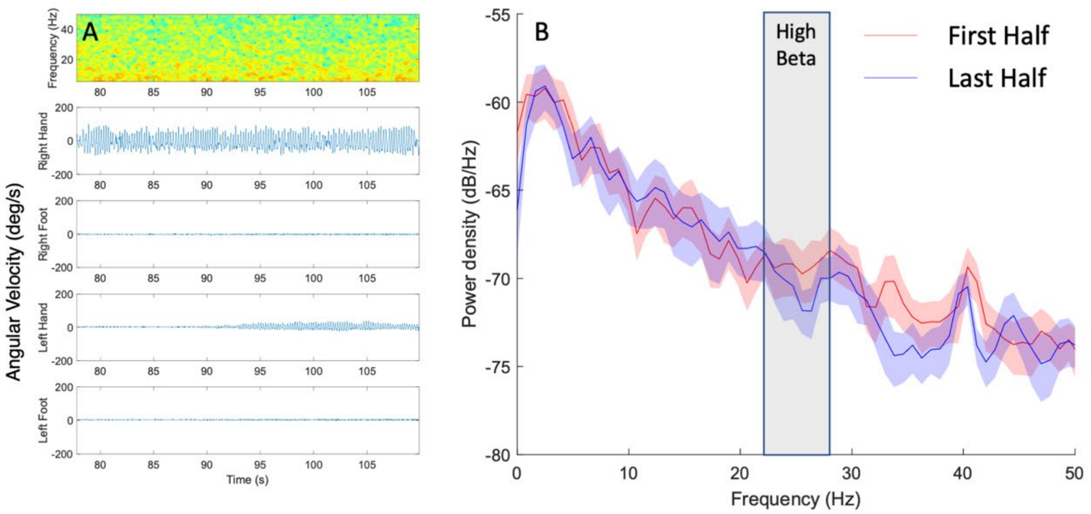

Figure S3. Example figure of Participant 6 (tremor-dominant) during the QDG task (right hand, left STN). **(A)** Synchronized spectrogram of neural data and movement of each limbed measured by a gyroscope. **(B)** Power spectral density plots of the first and last half of trial as there was no onset of tremor in the middle of the trial for the right body.

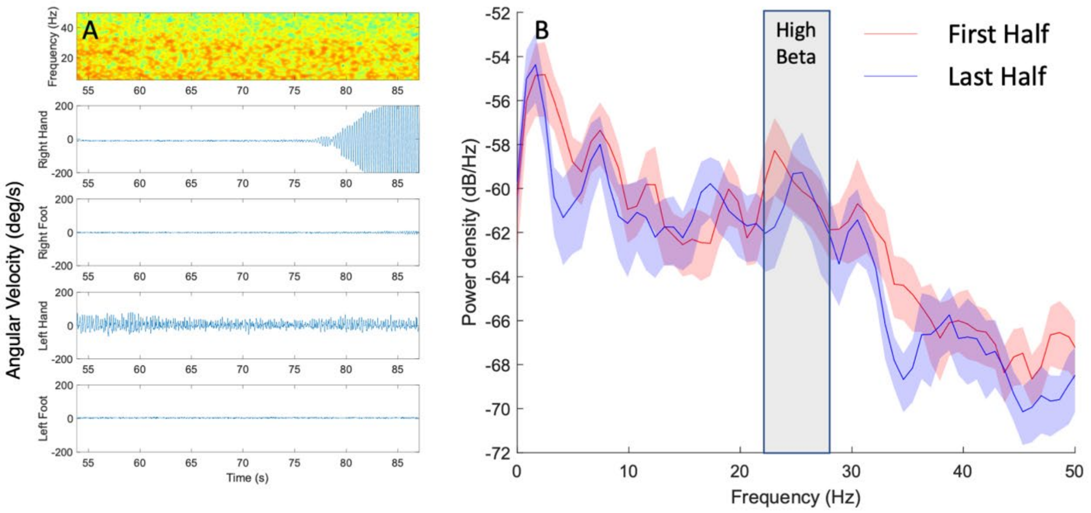

Figure S4. Example figure of Participant 6 (tremor-dominant) during the QDG task (left hand, right STN). **(A)** Synchronized spectrogram of neural data and movement of each limbed measured by a gyroscope. **(B)** Power spectral density plots of the first and last half of trial as there was no onset of tremor in the middle of the trial for the left body.

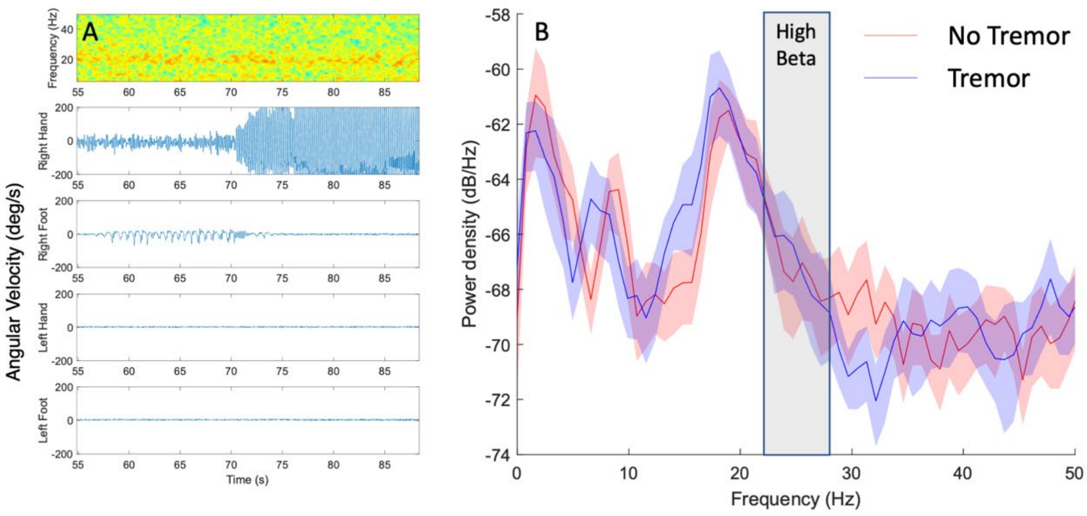

Figure S5. Example figure of Participant 8 (tremor-dominant) during the QDG task (right hand, left STN). **(A)** Synchronized spectrogram of neural data and movement of each limbed measured by a gyroscope. **(B)** Power spectral density plots of periods of no tremor and tremor in the right hand.

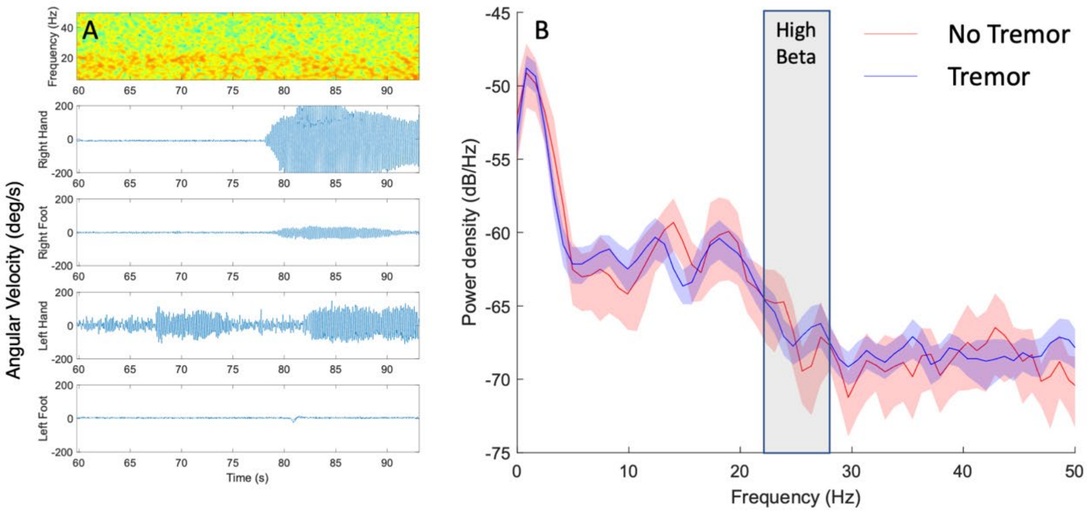

Figure S6. Example figure of Participant 8 (tremor-dominant) during the QDG task (left hand, right STN). **(A)** Synchronized spectrogram of neural data and movement of each limbed measured by a gyroscope. **(B)** Power spectral density plots of periods of no tremor and tremor in the left hand.

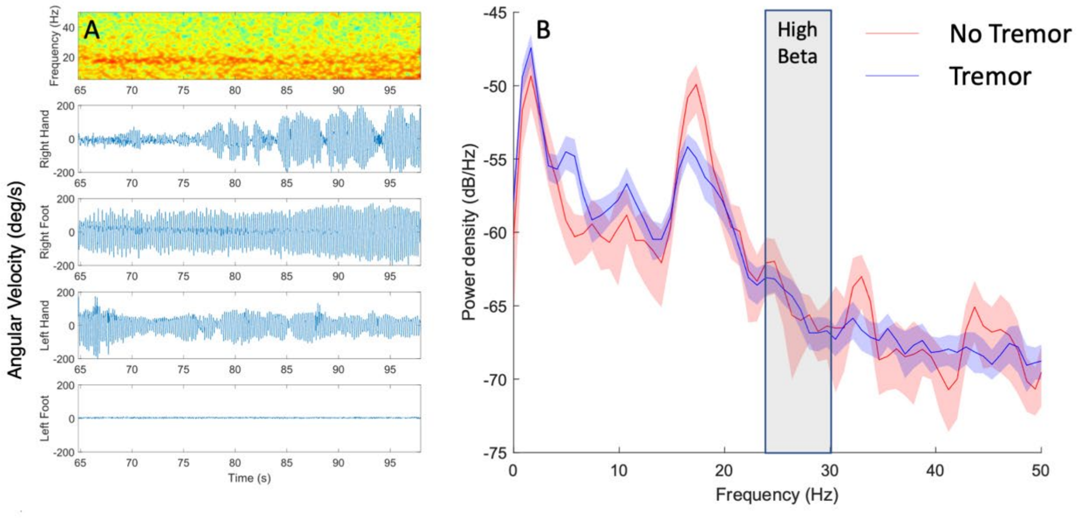

Figure S7. Example figure of Participant 12 (tremor-dominant) during the QDG task (right hand, left STN). **(A)** Synchronized spectrogram of neural data and movement of each limbed measured by a gyroscope. **(B)** Power spectral density plots of periods of no tremor and tremor in the right hand.

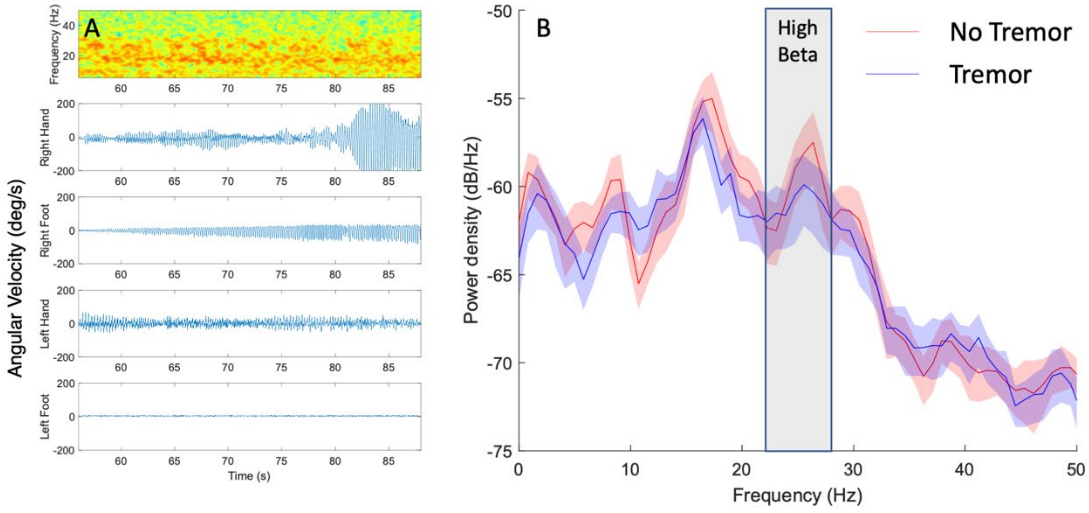

Figure S8. Example figure of Participant 12 (tremor-dominant) during the QDG task (left hand, right STN). **(A)** Synchronized spectrogram of neural data and movement of each limbed measured by a gyroscope. **(B)** Power spectral density plots of periods of no tremor and tremor in the left hand.
